# Supplementary material for: Implant-Supported Cantilever Fixed Partial Dentures in the Posterior Region: A Systematic Review and Meta-Analysis on Survival Outcomes
Source: Materials (Basel). 2025 Oct 14;18(20):4704. doi: 10.3390/ma18204704 (PMC12566088; doi:10.3390/ma18204704)
Supplement: Supplementary file 1 [file materials-18-04704-s001.zip › materials-3590582-supplementary.pdf]

SUPPLEMENTARY MATERIALS

***S1** - List of excluded articles*

Author	Year	Reference	Reason for exclusion
Al Kilani	2023	[1]	The studies investigated prostheses placed in both anterior (incisors to canines) and posterior regions. However, they did not provide a clear breakdown of failures and complications specific to each area (e.g., data presented in percentages), making it impossible to accurately assess survival rates or technical and biological complications related to posterior prostheses. Moreover, the available studies with published data may represent a sequential design, which further limits the ability to draw conclusions.
Becker	2004	[2]	
D’Albis	2022	[3]	
Horsch	2008	[4]	
Kim	2014	[5]	
Maló	2013	[6]	
Romeo	2003	[7]	
Romeo	2009	[8]	
Schmid	2020	[9]	
Fazel	2007	[10]	The studies did not specify the region (anterior/posterior) of the prostheses included in the respective samples.
Wennstrom	2004	[11]	
Shin	2008	[12]	Articles excluded for being written in languages other than those referred to in the inclusion criteria.
Mokhatari	2022	[12]	
Roccuzzo	2020	[13]	The study only followed prostheses in the anterior region.
Yang	2024	[14]	Cantilever extension < 3mm

1. Al-Kilani, J.; Al-Kilani, S.; Chrcanovic, B.R. Difference in marginal bone loss around implants between short implant-supported partial fixed prostheses with and without cantilever: a retrospective clinical study. *Int J Implant Dent*. **2023**, *9,* 46. https://doi.org/10.1186/s40729-023-00515-w.
2. Becker, C.M. Cantilever fixed prostheses utilizing dental implants: a 10-year retrospective analysis. *Quintessence Int*. **2004**, *35*, 437-441.
3. D’albis, G.; D’albis, V.; Susca, B.; Palma, M.; Krenawi, N.A. Implant-supported zirconia fixed partial dentures cantilevered in the lateral-posterior area: A 4-year clinical results. *Journal of Dental Research, Dental Clinics, Dental Prospects*. **2022**, *16*, 258-263. https://doi.org/10.34172/joddd.2022.041.
4. Horsch, L.; Kronsteiner, D.; Rammelsberg, P. Survival and complications o□ implant-supported cantilever fixed dental prostheses with zirconia and metal □rameworks: A retrospective cohort study. *Clin Implant Dent Relat Res*. **2022**, *24*, 621-629, https://doi.org/10.1111/cid.13125.
5. Kim, P.; Ivanovski, S.; Latcham, N.; Mattheos, N. The impact o□cantilevers on biological and technical success outcomes o□implant-supported fixed partial dentures. A retrospective cohort study. *Clin Oral Implants Res*. **2014**, *25*, 175-184. https://doi.org/10.1111/clr.12102.

6. Maló, P.; Nobre, M. A.; Lopes, A. The prognosis of partial implant-supported fixed dental prostheses with cantilevers. A 5-year retrospective cohort study. *Eur J Oral Implantol.* **2013**, *6*, 51-59.
7. Romeo, E.; Lops, D.; Margutti, E.; Ghisolfi, M.; Chiapasco, M.; Vogel, G. Implant-supported fixed cantilever prostheses in partially edentulous arches. A seven-year prospective study. *Clin Oral Implants Res.* **2003**, *14*, 303-311. https://doi.org/10.1034/j.1600-0501.2003.120905.x.
8. Romeo, E.; Tomasi, C.; Finini, I.; Casentini, P.; Lops, D. Implant-supported fixed cantilever prosthesis in partially edentulous jaws: a cohort prospective study. *Clin Oral Implants Res.* **2009**, *20*, 1278-1285, https://doi.org/10.1111/j.1600-0501.2009.01766.x.
9. Schmid, E.; Morandini, M.; Roccuzzo, A.; Ramseier, C.A.; Sculean, A.; Salvi, G.E. Clinical and radiographic outcomes of implant-supported fixed dental prostheses with cantilever extension. A retrospective cohort study with a follow-up of at least 10 years. *Clin Oral Implants Res.* **2020**, *31*, 1243-1252. https://doi.org/10.1111/clr.13672.
10. Fazel, A.; Rismanchian, M. Evaluation of Tissues Surrounding Implant Supported Fixed Partial Denture with and without Cantilever Extension. *Frontiers in Dentistry* **2007**, *4*, 160-164.
11. Wennström, J.; Zurdo, J.; Karlsson, S.; Ekestubbe, A.; Gröndahl, K.; Lindhe, J. Bone level change at implant-supported fixed partial dentures with and without cantilever extension after 5 years in function. *J Clin Periodontol.* **2004**, *31*, 1077-1083. https://doi.org/10.1111/j.1600-051X.2004.00603.x.
12. Solati, M.; Mokhtari, M.; Radvar, M. Evaluation of Alveolar Crestal Bone Loss around Cantilever-Based Implants and Comparison with Single-Tooth Implants. Journal of Mashhad Dental School. **2019**, *43*, 167-168. https://doi.org/10.22038/JMDS.2019.13106.
13. Roccuzzo, A.; Jensen, S.S.; Worsaae, N.; Gotfredsen, K. Implant-supported 2-unit cantilevers compared with single crowns on adjacent implants: A comparative retrospective case series. *J Prosthet Dent.* **2020**, *123*, 717-723. https://doi.org/10.1016/j.prosdent.2019.04.024.
14. Yang, Y.; Gao, J.; Man, Y.; Yang, X.; Wu, Y.; Xiang, L.; Qu, Y. Effect of the mesiodistal cantilever on implant-supported single crowns on biological and technical complications: A retrospective study. *Int J Oral Implantol (Berl).* **2024**, *17*, 383-400.

S2. *Risk of bias of cohort studies*

Assessment quality of Non-RCT included studies based on New Castle Ottawa (cohort studies).

Studies	Selection				Comparability		Outcome			Total
	**Exposed Cohort**	**Non exposited cohort**	**Ascertainment of exposure**	**Outcome of interest not present at start**	**Main Factor**	**Additional Factor**	**Assessment of outcome**	**Follow-up long enough***	**Adequacy of follow-up of cohorts**	
Aglietta et al. 2012	☆	--	☆	---	☆	☆	☆	☆	☆	7
Dereci et al. 2021	☆	☆	☆	☆	☆	☆	☆	☆	☆	9
Halg et al. 2008	☆	☆	☆	☆	☆	☆	☆	☆	☆	9
Jensen-Louwerse et al. 2021	☆	--	☆	--	☆	☆	☆	☆	☆	7
Kim et al. 2018	☆	--	☆	---	☆	☆	☆	☆	☆	7
Palmer et al. 2011	☆	--	☆	---	☆	☆	☆	☆	☆	7
Roccuzzo et al. 2023	☆	--	☆	---	☆	☆	☆	☆	☆	7
Schmid et al. 2021	☆	--	☆	---	☆	☆	☆	☆	☆	7

*Six months was considered an adequate follow-up period;

S3. *Risk of bias of case-control studies*

Assessment quality of Non-RCT included studies based on New Castle Ottawa (case control studies).

Studies	Selection				Comparability	Exposure			Total
	**Definition adequate**	**Representativeness**	**Controls**	**Definition of controls**	**design**	**Ascertaiment of exposure**	**Method**	**Non-response rate**	
Cannizaro et al. 2020	☆	☆	--	--	☆	☆	--	--	4

*Cannizzaro et al. 2020: without control group.

S4. *Summary of findings and details of the GRADE assessment for the main outcomes of this systematic review.*

Patients: Individuals requiring oral rehabilitation with implants;

Intervention: Rehabilitative treatment with implant-supported fixed partial dentures (FPDs) with cantilevers in the posterior region;

Comparison: Patients rehabilitated with implant-supported fixed partial dentures without cantilevers in the posterior region;

Outcome: Evaluation results of implant and prosthesis survival rates, technical and biological complications, and marginal bone loss for both rehabilitation methods.

Certainty assessment							Summary of findings					Importance
							№ of patients		Effect		Certainty	
№ of studies	Study design	Risk of bias	Inconsistency	Indirectness	Imprecision	Other considerations	Failure dental implants/Prostheses		Relative (95% CI)	Absolute (95% CI)		
**New outcome Failure of implant placement in cantilever prostheses (follow-up: mean 61 months; assessed with: Units)**												
10	non-randomised studies	serious [a]	not serious	not serious	not serious	publication bias strongly suspected [b]	7/293	7/230	**Rate ratio 0.01** (0.00 to 0.03)	**0.01 per 100 patient (s)** (from 0.00 to 0.03)	⊕◯◯◯ Very low [a,b]	CRITICAL
**Failure of implant-supported prostheses with cantilever (follow-up: mean 61 months; assessed with: units.)**												
9	non-randomised studies	serious [a]	not serious	not serious	not serious	publication bias strongly suspected [b]	4/226	4/209	**Rate ratio 0.01** (0.00 to 0.03)	**0.01 per 100 patient (s)** (from 0.00 to 0.03)	⊕◯◯◯ Very low [a,b]	CRITICAL
**Complications involving the prosthesis and/or prosthetic abutment (assessed with: units)**												

Certainty assessment							Summary of findings					Importance
№ of studies	Study design	Risk of bias	Inconsistency	Indirectness	Imprecision	Other considerations	№ of patients		Effect		Certainty	
							Failure dental implants/Prostheses		Relative (95% CI)	Absolute (95% CI)		
4	non-randomised studies	serious [a]	serious [c]	not serious	serious [b,d]	publication bias strongly suspected [e]	22/143	22/137	**Rate ratio 0.14** (0.05 to 0.26)	**0.14 per 100 patient(s)** (from 0.05 to 0.26)	⨁◯◯◯ Very low [a,b,c,d,e]	CRITICAL

Prosthetic retention loss (assessed with: unit)

№ of studies	Study design	Risk of bias	Inconsistency	Indirectness	Imprecision	Other considerations	Failure dental implants/Prostheses		Relative (95% CI)	Absolute (95% CI)	Certainty	Importance
7	non-randomised studies	serious [a]	not serious	not serious	not serious	publication bias strongly suspected [b]	25/177	25/171	**Rate ratio 0.13** (0.07 to 0.21)	**0.13 per 100 patient(s)** (from 0.07 to 0.21)	⨁◯◯◯ Very low [a,b]	CRITICAL

Technical complications in relation to cantilever length (assessed with: Units)

№ of studies	Study design	Risk of bias	Inconsistency	Indirectness	Imprecision	Other considerations	Failure dental implants/Prostheses		Relative (95% CI)	Absolute (95% CI)	Certainty	Importance
6	non-randomised studies	serious [a]	serious [c]	not serious	serious [d]	publication bias strongly suspected [e]	43/143	43/147	**Rate ratio 0.29** (0.16 to 0.43)	**0.29 per 100 patient(s)** (from 0.16 to 0.43)	⨁◯◯◯ Very low [a,c,d,e]	CRITICAL

Biological complications in ISPDCs (general analysis and study design)

№ of studies	Study design	Risk of bias	Inconsistency	Indirectness	Imprecision	Other considerations	Failure dental implants/Prostheses		Relative (95% CI)	Absolute (95% CI)	Certainty	Importance
~~7~~6	non-randomised studies	serious [a]	serious [c]	not serious	serious [d]	publication bias strongly suspected [e]	41/175	41/142	**Rate ratio 0.20** (0.04 to 0.43)	**0.20 per 100 patient(s)** (from 0.04 to 0.43)	⨁◯◯◯ Very low [a,c,d,e]	CRITICAL

Marginal bone loss (final timepoint) stratified by study design (assessed with: mm)

Certainty assessment							Summary of findings					
№ of studies	Study design	Risk of bias	Inconsistency	Indirectness	Imprecision	Other considerations	№ of patients		Effect		Certainty	Importance
							Failure dental implants/Prostheses		Relative (95% CI)	Absolute (95% CI)		
7	non-randomised studies	serious [a]	serious [c]	not serious	not serious	publication bias strongly suspected all plausible residual confounding would reduce the demonstrated effect [b]	153	153	-	mean **0.85 1.31 higher** (0.4 higher to 1.31 higher)	⊕◯◯◯ Very low [a,b,c]	CRITICAL
**Change in marginal bone loss from baseline to final timepoint (TF–T0) (assessed with: mm)**												
4	non-randomised studies	serious [a]	serious [c]	not serious	not serious	publication bias strongly suspected all plausible residual confounding would reduce the demonstrated effect [b]	89	93	-	MD **0.35 0.66 higher** (0.04 higher to 0.66 higher)	⊕◯◯◯ Very low [a,b,c]	CRITICAL
**Marginal bone loss at adjacent versus distant implant sites (assessed with: mm)**												
4	non-randomised studies	serious [a]	not serious	not serious	not serious	publication bias strongly suspected all plausible residual confounding would reduce the demonstrated effect [b]	109	109	-	MD **0.01 0.16 higher** (0.15 lower to 0.16 higher)	⊕◯◯◯ Very low [a,b]	CRITICAL
**Complication rates comparing screw-retained and cemented-retained prostheses (assessed with: Units.)**												
4	non-randomised studies	serious [a]	serious [c]	not serious	serious [b]	publication bias strongly suspected [b]	19/108	19/129	**Rate ratio 0.16** (0.04 to 0.32)	**0.16 per 100 patient(s) per years** (from 0.04 to 0.32)	⊕◯◯◯ Very low [a,b,c]	CRITICAL
**Retention rates comparing screw-retained and cement-retained prostheses (assessed with: Units.)**												

Certainty assessment							Summary of findings					Importance
							№ of patients		Effect			
№ of studies	Study design	Risk of bias	Inconsistency	Indirectness	Imprecision	Other considerations	Failure dental implants/Prostheses		Relative (95% CI)	Absolute (95% CI)	Certainty	
5	non-randomised studies	serious [a]	serious [c]	not serious	serious [b]	publication bias strongly suspected [b,e]	18/126	18/147	**Rate ratio 0.13** (0.05 to 0.24)	**0.13 per 100 patient(s) per years** (from 0.05 to 0.24)	⨁◯◯◯ Very low [a,b,c,e]	CRITICAL
**Technical complications in ISPDCs, according to the type of occlusal veneering material (assessed with: Units)**												
~~6~~5	non-randomised studies	serious [a]	serious [c]	not serious	serious [b]	publication bias strongly suspected [b]	37/126	37/147	**Rate ratio 0.28** (0.14 to 0.45)	**0.28 per 100 patient(s) per years** (from 0.14 to 0.45)	⨁◯◯◯ Very low [a,b,c]	CRITICAL

a - Random sequence generation and allocation concealment
b – Small sample size or absence of sample size calculation.
c – There is significant heterogeneity, with I^2 values greater than 40%.
d – non-overlapping confidence intervals
e - The number of studies for this outcome has been reduced.

S5. Summary of main outcomes of this systematic review

Outcome	Anticipated absolute effects (95% CI)		Relative effect (95% CI)	№ of participants (studies)	Certainty
	Risk with	Risk with Failure dental implants/Prostheses			
**New outcome Failure of implant placement in cantilever prostheses (Failure of implant placement in cantilever prostheses) assessed with: Units follow-up: mean 61 months**	3 per 100	**0 per 100** (0 to 0)	**Rate ratio 0.01** (0.00 to 0.03)	523 (10 non-randomised studies)	⊕◯◯◯ Very low [a,b]
**Failure of implant-supported prostheses with cantilever (cantilever) assessed with: units. follow-up: mean 61 months**	2 per 100	**0 per 100** (0 to 0)	**Rate ratio 0.01** (0.00 to 0.03)	435 (9 non-randomised studies)	⊕◯◯◯ Very low [a,b]
**Complications involving the prosthesis and/or prosthetic abutment assessed with: units**	16 per 100	**2 per 100** (1 to 4)	**Rate ratio 0.14** (0.05 to 0.26)	280 (4 non-randomised studies)	⊕◯◯◯ Very low [a,b,c,d,e]
**Prosthetic retention loss (Prosthetic retention loss) assessed with: unit**	15 per 100	**2 per 100** (1 to 3)	**Rate ratio 0.13** (0.07 to 0.21)	348 (7 non-randomised studies)	⊕◯◯◯ Very low [a,b]
**Technical complications in relation to cantilever length assessed with: Units**	29 per 100	**8 per 100** (5 to 13)	**Rate ratio 0.29** (0.16 to 0.43)	290 (6 non-randomised studies)	⊕◯◯◯ Very low [a,c,d,e]
**Biological complications in ISPDCs (general analysis and study design)**	29 per 100	**6 per 100** (1 to 12)	**Rate ratio 0.20** (0.04 to 0.43)	317 (7 non-randomised studies)	⊕◯◯◯ Very low [a,c,d,e]
**Marginal bone loss (final timepoint) stratified by study design assessed with: mm**	The mean marginal bone loss (final timepoint) stratified by study design was **0** 1.31	mean **0.85 1.31 higher** (0.4 higher to 1.31 higher)	-	306 (7 non-randomised studies)	⊕◯◯◯ Very low [a,b,c]
**Change in marginal bone loss from baseline to final timepoint (TF–T0) assessed with: mm**	The mean change in marginal bone loss from baseline to final timepoint (TF–T0) was **0** 0.66	MD **0.35 0.66 higher** (0.04 higher to 0.66 higher)	-	182 (4 non-randomised studies)	⊕◯◯◯ Very low [a,b,c]
**Marginal bone loss at adjacent versus distant implant sites assessed with: mm**	The mean marginal bone loss at adjacent versus distant implant sites was **0** 0.16	MD **0.01 0.16 higher** (0.15 lower to 0.16 higher)	-	218 (4 non-randomised studies)	⊕◯◯◯ Very low [a,b]
**Complication rates comparing screw-retained and cemented-retained prostheses (screw-retained and cemented-retained prostheses) assessed with: Units.**	15 per 100	**2 per 100** (1 to 5)	**Rate ratio 0.16** (0.04 to 0.32)	237 (4 non-randomised studies)	⊕◯◯◯ Very low [a,b,c]
**Retention rates comparing screw-retained and cement-retained prostheses assessed with: Units.**	12 per 100	**2 per 100** (1 to 3)	**Rate ratio 0.13** (0.05 to 0.24)	273 (5 non-randomised studies)	⊕◯◯◯ Very low [a,b,c,e]

Outcome	Anticipated absolute effects (95% CI)		Relative effect (95% CI)	№ of participants (studies)	Certainty
	Risk with	Risk with Failure dental implants/Prostheses			
**Technical complications in ISPDCs, according to the type of occlusal veneering material (occlusal veneering material) assessed with: Units**	25 per 100	**7 per 100** (4 to 11)	**Rate ratio 0.28** (0.14 to 0.45)	273 (6 non-randomised studies)	⊕◯◯◯ Very low [a,b,c]

PRISMA-P (Preferred Reporting Items for Systematic review and Meta-Analysis Protocols) 2015 checklist: recommended items to address in a systematic review protocol*

Section and topic	Item No	Checklist item	Page
**ADMINISTRATIVE INFORMATION**			
Title:			
Identification	1a	Identify the report as a protocol of a systematic review	CRD42024606201
Update	1b	If the protocol is for an update of a previous systematic review, identify as such	None.
Registration	2	If registered, provide the name of the registry (such as PROSPERO) and registration number	CRD42024606201
Authors:			
Contact	3a	Provide name, institutional affiliation, e-mail address of all protocol authors; provide physical mailing address of corresponding author	1
Contributions	3b	Describe contributions of protocol authors and identify the guarantor of the review	13
Amendments	4	If the protocol represents an amendment of a previously completed or published protocol, identify as such and list changes; otherwise, state plan for documenting important protocol amendments	CRD42024606201
Support:			
Sources	5a	Indicate sources of financial or other support for the review	13
Sponsor	5b	Provide name for the review funder and/or sponsor	13
Role of sponsor or funder	5c	Describe roles of funder(s), sponsor(s), and/or institution(s), if any, in developing the protocol	13
**INTRODUCTION**			
Rationale	6	Describe the rationale for the review in the context of what is already known	1-2
Objectives	7	Provide an explicit statement of the question(s) the review will address with reference to participants, interventions, comparators, and outcomes (PICO)	2
**METHODS**			

Eligibility criteria	8	Specify the study characteristics (such as PICO, study design, setting, time frame) and report characteristics (such as years considered, language, publication status) to be used as criteria for eligibility for the review	2
Information sources	9	Describe all intended information sources (such as electronic databases, contact with study authors, trial registers or other grey literature sources) with planned dates of coverage	2
Search strategy	10	Present draft of search strategy to be used for at least one electronic database, including planned limits, such that it could be repeated	2
Study records:			
Data management	11a	Describe the mechanism(s) that will be used to manage records and data throughout the review	2
Selection process	11b	State the process that will be used for selecting studies (such as two independent reviewers) through each phase of the review (that is, screening, eligibility and inclusion in meta-analysis)	2
Data collection process	11c	Describe planned method of extracting data from reports (such as piloting forms, done independently, in duplicate), any processes for obtaining and confirming data from investigators	2-3
Data items	12	List and define all variables for which data will be sought (such as PICO items, funding sources), any pre-planned data assumptions and simplifications	2-3
Outcomes and prioritization	13	List and define all outcomes for which data will be sought, including prioritization of main and additional outcomes, with rationale	3
Risk of bias in individual studies	14	Describe anticipated methods for assessing risk of bias of individual studies, including whether this will be done at the outcome or study level, or both; state how this information will be used in data synthesis	3
Data synthesis	15a	Describe criteria under which study data will be quantitatively synthesised	3
	15b	If data are appropriate for quantitative synthesis, describe planned summary measures, methods of handling data and methods of combining data from studies, including any planned exploration of consistency (such as I^2, Kendall's τ)	3
	15c	Describe any proposed additional analyses (such as sensitivity or subgroup analyses, meta-regression)	3
	15d	If quantitative synthesis is not appropriate, describe the type of summary planned	None.
Meta-bias(es)	16	Specify any planned assessment of meta-bias(es) (such as publication bias across studies, selective reporting within studies)	3

Confidence in cumulative evidence	17	Describe how the strength of the body of evidence will be assessed (such as GRADE)	3

*** It is strongly recommended that this checklist be read in conjunction with the PRISMA-P Explanation and Elaboration (cite when available) for important clarification on the items. Amendments to a review protocol should be tracked and dated. The copyright for PRISMA-P (including checklist) is held by the PRISMA-P Group and is distributed under a Creative Commons Attribution Licence 4.0.**

From: Shamseer L, Moher D, Clarke M, Ghersi D, Liberati A, Petticrew M, Shekelle P, Stewart L, PRISMA-P Group. Preferred reporting items for systematic review and meta-analysis protocols (PRISMA-P) 2015: elaboration and explanation. BMJ. 2015 Jan 2;349(jan02 1):g7647.

PRISMA 2020 Checklist

Section and Topic	Item #	Checklist item	Location where item is reported (page)
**TITLE**			
Title	1	Identify the report as a systematic review.	1
**ABSTRACT**			
Abstract	2	See the PRISMA 2020 for Abstracts checklist.	1
**INTRODUCTION**			
Rationale	3	Describe the rationale for the review in the context of existing knowledge.	1-2
Objectives	4	Provide an explicit statement of the objective(s) or question(s) the review addresses.	2
**METHODS**			
Eligibility criteria	5	Specify the inclusion and exclusion criteria for the review and how studies were grouped for the syntheses.	2
Information sources	6	Specify all databases, registers, websites, organisations, reference lists and other sources searched or consulted to identify studies. Specify the date when each source was last searched or consulted.	2
Search strategy	7	Present the full search strategies for all databases, registers and websites, including any filters and limits used.	2
Selection process	8	Specify the methods used to decide whether a study met the inclusion criteria of the review, including how many reviewers screened each record and each report retrieved, whether they worked independently, and if applicable, details of automation tools used in the process.	2
Data collection process	9	Specify the methods used to collect data from reports, including how many reviewers collected data from each report, whether they worked independently, any processes for obtaining or confirming data from study investigators, and if applicable, details of automation tools used in the process.	2-3
Data items	10a	List and define all outcomes for which data were sought. Specify whether all results that were compatible with each outcome domain in each study were sought (e.g. for all measures, time points, analyses), and if not, the methods used to decide which results to collect.	2-3
	10b	List and define all other variables for which data were sought (e.g. participant and intervention characteristics, funding sources). Describe any assumptions made about any missing or unclear information.	2-3
Study risk of bias assessment	11	Specify the methods used to assess risk of bias in the included studies, including details of the tool(s) used, how many reviewers assessed each study and whether they worked independently, and if applicable, details of automation tools used in the process.	3
Effect measures	12	Specify for each outcome the effect measure(s) (e.g. risk ratio, mean difference) used in the synthesis or presentation of results.	3
Synthesis methods	13a	Describe the processes used to decide which studies were eligible for each synthesis (e.g. tabulating the study intervention characteristics and comparing against the planned groups for each synthesis (item #5)).	3
	13b	Describe any methods required to prepare the data for presentation or synthesis, such as handling of missing summary statistics, or data conversions.	3
	13c	Describe any methods used to tabulate or visually display results of individual studies and syntheses.	3

Section and Topic	Item #	Checklist item	Location where item is reported (page)
	13d	Describe any methods used to synthesize results and provide a rationale for the choice(s). If meta-analysis was performed, describe the model(s), method(s) to identify the presence and extent of statistical heterogeneity, and software package(s) used.	3
	13e	Describe any methods used to explore possible causes of heterogeneity among study results (e.g. subgroup analysis, meta-regression).	3
	13f	Describe any sensitivity analyses conducted to assess robustness of the synthesized results.	3
Reporting bias assessment	14	Describe any methods used to assess risk of bias due to missing results in a synthesis (arising from reporting biases).	3
Certainty assessment	15	Describe any methods used to assess certainty (or confidence) in the body of evidence for an outcome.	3
**RESULTS**			
Study selection	16a	Describe the results of the search and selection process, from the number of records identified in the search to the number of studies included in the review, ideally using a flow diagram.	3-4
	16b	Cite studies that might appear to meet the inclusion criteria, but which were excluded, and explain why they were excluded.	3-4
Study characteristics	17	Cite each included study and present its characteristics.	4-5
Risk of bias in studies	18	Present assessments of risk of bias for each included study.	10
Results of individual studies	19	For all outcomes, present, for each study: (a) summary statistics for each group (where appropriate) and (b) an effect estimate and its precision (e.g. confidence/credible interval), ideally using structured tables or plots.	Table 1
Results of syntheses	20a	For each synthesis, briefly summarise the characteristics and risk of bias among contributing studies.	10-11
	20b	Present results of all statistical syntheses conducted. If meta-analysis was done, present for each the summary estimate and its precision (e.g. confidence/credible interval) and measures of statistical heterogeneity. If comparing groups, describe the direction of the effect.	6-10
	20c	Present results of all investigations of possible causes of heterogeneity among study results.	6-10
	20d	Present results of all sensitivity analyses conducted to assess the robustness of the synthesized results.	6-10
Reporting biases	21	Present assessments of risk of bias due to missing results (arising from reporting biases) for each synthesis assessed.	10-11
Certainty of evidence	22	Present assessments of certainty (or confidence) in the body of evidence for each outcome assessed.	10-11
**DISCUSSION**			
Discussion	23a	Provide a general interpretation of the results in the context of other evidence.	11

Section and Topic	Item #	Checklist item	Location where item is reported (page)
	23b	Discuss any limitations of the evidence included in the review.	13
	23c	Discuss any limitations of the review processes used.	13
	23d	Discuss implications of the results for practice, policy, and future research.	13
**OTHER INFORMATION**			
Registration and protocol	24a	Provide registration information for the review, including register name and registration number, or state that the review was not registered.	1
	24b	Indicate where the review protocol can be accessed, or state that a protocol was not prepared.	2
	24c	Describe and explain any amendments to information provided at registration or in the protocol.	2
Support	25	Describe sources of financial or non-financial support for the review, and the role of the funders or sponsors in the review.	13
Competing interests	26	Declare any competing interests of review authors.	13
Availability of data, code and other materials	27	Report which of the following are publicly available and where they can be found: template data collection forms; data extracted from included studies; data used for all analyses; analytic code; any other materials used in the review.	13

From: Page MJ, McKenzie JE, Bossuyt PM, Boutron I, Hoffmann TC, Mulrow CD, et al. The PRISMA 2020 statement: an updated guideline for reporting systematic reviews. BMJ 2021;372:n71. doi: 10.1136/bmj.n71. This work is licensed under CC BY 4.0. To view a copy of this license, visit https://creativecommons.org/licenses/by/4.0/
